# Comparative Evaluation of the Effects of Consumption of Colombian Agraz (*Vaccinium meridionale* Swartz) on Insulin Resistance, Antioxidant Capacity, and Markers of Oxidation and Inflammation, Between Men and Women with Metabolic Syndrome

**DOI:** 10.1089/biores.2020.0053

**Published:** 2020-11-30

**Authors:** Yeisson Galvis-Pérez, Catalina Marín-Echeverri, Claudia Patricia Franco Escobar, Juan C. Aristizábal, Maria-Luz Fernández, Jacqueline Barona-Acevedo

**Affiliations:** ^1^ Research Group of Toxinology, Therapeutic and Food Alternatives, School of Microbiology, Universidad de Antioquia UdeA, Medellín, Colombia.; ^2^ Research Group of Physiology and Biochemistry (PHYSIS), School of Nutrition and Dietetics, Universidad de Antioquia UdeA, Medellín, Colombia.; ^3^ Department of Nutritional Sciences, University of Connecticut, Storrs, Connecticut, USA.

**Keywords:** agraz, antioxidants, inflammation, insulin resistance, metabolic syndrome, oxidative stress

## Abstract

The metabolic syndrome (MS) is a constellation of related factors that increases the risk of developing cardiovascular diseases. *Vaccinium meridionale* Swartz contains polyphenols that could modulate some components of MS. Epidemiological and intervention studies have shown differences between men and women in MS components and antioxidant capacity. The objective of this study is to compare between men and women with MS the effects of agraz consumption on insulin resistance, antioxidant capacity, and markers of oxidation and inflammation. Men and women diagnosed with MS according to the Adult Treatment Panel III criteria were recruited in a double-blind, crossover study of 12 weeks. Participants were assigned to consume agraz nectar or placebo over 4 weeks. After 4 weeks of washout, they were switched to the alternative treatment. At the end of each period, the components of the MS, insulin resistance, antioxidant capacity, and some oxidative (oxidized low-density lipoprotein [oxLDL]; thiobarbituric acid reactive substances) and inflammatory (high-sensitive C-reactive protein [hs-CRP]) markers were evaluated. After consuming agraz, there was a tendency to increase the levels of antioxidants and to reduce the levels of hs-CRP in both genders. In addition, women who increased their serum phenols after consuming agraz had a significant reduction in insulin resistance, which was different from the results in men. Regarding men, those who increased their serum antioxidant capacity after consuming agraz had a better effect on the reduction of oxLDL levels that was significant compared to women. There are important differences between genders in the effects of agraz consumption in adults with MS.

## Introduction

Metabolic syndrome (MS) increases the risk of developing type 2 diabetes and cardiovascular diseases.^1,2^ The prevalence of MS oscillates between 14% and 25% in adults in most countries.^3^ In Colombia, the prevalence of MS oscillates according to the region of the country and the population studied, with values ranging from 17%^4^ to 41%.^5^

In 1988, Reaven postulated insulin resistance as a central event in the pathogenesis of MS.^6^ In addition to insulin resistance, there is accumulation of abdominal fat, high glucose levels, and high blood pressure (BP).^2,7^ These alterations can generate a pro-inflammatory state^8,9^ and oxidative stress caused by the increase in reactive oxygen species (ROS).^8,10^

In healthy people there is a balance between ROS production and antioxidant agents capable of neutralizing ROS, but in patients with MS there is a reduction in antioxidant (endogenous) defenses and a greater increase in ROS^7,11^; this imbalance has been named as oxidative stress. Therefore, the increase in the consumption of antioxidants has been proposed as a therapeutic alternative for patients with MS.^12^ Fruits and vegetables contain numerous polyphenols, which have shown not only antioxidant but also anti-inflammatory and lipid-lowering activities, presenting a great potential to improve the risk factors for MS, with fewer adverse effects.^12–14^

Colombia is rich in fruits and vegetables. An example is *Vaccinium meridionale* Swartz or agraz, a purple fruit of the *Vaccinium* family, of which 400 different species have been registered, rich in polyphenolic compounds with antioxidant properties,^15,16^ which have been profiled as potential candidates to reduce oxidative stress^17–19^ and the pro-inflammatory state seen in MS.^20^ Studies in other countries, evaluating the effects of blueberry (*Vaccinium ashei* and *Vaccinium corymbosum*) consumption in humans with MS and obesity, observed decreases in insulin resistance and biomarkers of lipid peroxidation and inflammation.^21,22^ Likewise, studies conducted with the Colombian agraz showed positive effects in markers of inflammation and lipid function in women with this syndrome.^23,24^

To evaluate the effects of polyphenol-rich fruit consumption on MS components, it is important to consider the characteristics of the population studied, given that epidemiological and clinical studies have recommended different cutoff points or risk values for MS according to gender.^25–29^ Likewise, several studies found differences between men and women in antioxidant capacity after consuming fruits,^30–33^ but these studies did not include people with MS nor have studied differential effects between men and women after consuming agraz for this variable or other related to MS components. Therefore, this study determined the effects of consuming the Colombian agraz on insulin resistance, antioxidant capacity, and markers of inflammation and oxidation between men and women with MS.

## Materials and Methods

### Study population

We included a population of 26 men and 26 women with MS. To the authors knowledge no previous studies with *Vaccinium* have reported or analyzed differences between men and women. Therefore, sample size estimation was based on previous studies in people with MS consuming a polyphenol-rich fruit (grapes) following a similar protocol, in which a significant reduction on BP, triglycerides, and some inflammatory markers was observed in each gender separately.^34,35^ In fact, a smaller sample size has been sufficient to observe significant differences on markers of oxidative stress in people with cardiovascular risk factors in a previous crossover design intervention with *Vaccinium*.^36^ All participants diagnosed with MS according to the Adult Treatment Panel III criteria^1^ were paired according to age and body mass index. Exclusion criteria included kidney disease, heart disease, taking any anti-inflammatory, antihypertensive, lipid-lowering medication, supplements, or nutraceuticals, and cigarette smoking.

The study was carried out in accordance with the Code of Ethics of the World Medical Association (Declaration of Helsinki) for experiments with humans. The Bioethics Committee for Human Research of the University Research Headquarters of the University of Antioquia approved the study (Act number 17-58-746, 2017). The study was classified as a minimum risk investigation in accordance with the Resolution No. 8430 of the Colombian Ministry of Health.

### Experimental design

This was a double blind, crossover design study, lasting 12 weeks, where the effects of agraz and placebo on MS variables, markers of oxidative stress, inflammation, antioxidant capacity, and insulin resistance were compared between men and women. Subjects were recruited from the University of Antioquia, Medellín-Colombia by flyers, massive emails, and voice-to-voice. After the baseline visit to diagnose *de novo* MS, the volunteers were assigned to consume agraz nectar or placebo over 4 weeks; after 4 weeks of washout they were switched to the alternative treatment. Participants made four visits at the Research and Clinical Laboratory from the School of Microbiology, University of Antioquia at the beginning and end of each consumption period (agraz and placebo) to measure anthropometric variables, BP, and to obtain blood samples to measure the biochemical variables. Participants were asked to maintain their body weight, usual physical activity level, and dietary habits, except for the consumption of polyphenol-rich foods during the whole study, including the washout period. There was constant communication with the participants to deliver the beverages (agraz or placebo) every 3 days, to measure body weight and compliance using a weekly questionnaire, and to know possible adverse effects. In case of any adverse effect, subjects were instructed to visit their family physician and to report to the research team to evaluate their continuity in the study.

In addition, subjects registered their diet (using a food frequency questionnaire) and a 7-day physical activity record at baseline and last week of each period to verify no changes in diet or exercise.

### Nectar of agraz and placebo

Agraz fruits were processed and lyophilized as previously described.^37^ The lyophilized fruit was reconstituted to provide an agraz nectar. The daily dose was equivalent to the total phenols present in 200 g of fresh fruit. The placebo was elaborated to resemble the sensory aspects and macronutrients of the agraz nectar, except for the content of polyphenols.

### Blood sampling

Blood was extracted after 10–12 h of overnight fasting, from the antecubital vein in ethylenediaminetetraacetic acid tubes and in tubes without anticoagulant to obtain serum. The samples were stored at −70°C for subsequent analysis.

### Isolation of peripheral blood mononuclear cells

Peripheral blood mononuclear cells were isolated using a density gradient with the Histopaque 1077 reagent (Sigma-Aldrich, St. Louis, MO) as previously described.^38^

### Blood pressure

The armband of an automatic BP monitor (Omron, Healthcare, Inc., Bannockburn, IL) was positioned in the right arm at the level of the heart, after 5 min of resting in the sitting position. Two measurements separate by 1 min were made, if the two values were different in >5 mmHg, a third measurement was done, and the average was used for the analysis.

### Anthropometric measures

Body weight (kg) was obtained on a calibrated digital scale. The abdominal perimeter was measured in cm at the upper edge of the iliac crest using a flexible (nonelastic) body tape after a normal expiration.

### Blood glucose, triglycerides, and high-density lipoprotein cholesterol

These biochemical variables were measured by enzymatic-colorimetric methods using an automated analyzer (SIEMENS Dimension Clinical Chemistry System, Germany).

### Insulin and homeostatic model assessment of insulin resistance

Insulin was measured by enzyme-linked immunosorbent assay (ELISA; ADVIA Centaur^®^ CP) using an automated analyzer (SIEMENS Dimension Clinical Chemistry System). Homeostatic model assessment of insulin resistance (HOMA-IR) was calculated with the following formula: HOMAIR = (insulin μUI/mL × glucose mg/dL)/405.^39^

### Inflammatory markers

High-sensitive C-reactive protein (hs-CRP) was measured by immunoturbidimetry in an automatic analyzer (SIEMENS Dimension Clinical Chemistry System). Nuclear factor enhancer of the kappa light chains of activated B cells (NFκB) transcription factor was measured by a sandwich ELISA with the NFκB p65 Transcription Factor Assay Kit (ab176648; abcam, USA), which quantifies the p65 fraction in nuclear extracts of mononuclear cells.

### Oxidative markers

Thiobarbituric acid reactive substances (TBARS) were determined using the TBARS Assay Kit (Cayman Chemical, USA) by spectrophotometry at 530 nm. The Malondialdehyde (MDA) content of the unknown samples was determined with a standard MDA curve of known concentrations. Results were expressed as μM MDA. Oxidized low-density lipoprotein (oxLDL) was measured using the Human Oxidized LDL ELISA Kit (MDA LDL, Catalog number STA-369) following the manufacturer's instructions (Cell Biolabs, Inc., San Diego, CA).

### Total phenols and antioxidant capacity measured by ferric reducing antioxidant power

To eliminate the interference of proteins for these measurements, the participant’ serum was deproteinized following the protocol described by Serafini et al.^40^ with some modifications. Total phenolic concentration in deproteinized serum was measured by the Folin–Ciocalteu method.^41^ The assays were performed by triplicate, and the results expressed as mg of Gallic Acid per liter. The serum iron reduction ability was measured following the procedure described by Benzie and Strain^42^ with some modifications.

### Statistical analyses

For the quantitative variables, summary measures were estimated, and for the qualitative variables, measures of absolute and relative frequency were obtained. For the variables with four measurement times, an analysis of variance (ANOVA) of repeated measurements was performed, and for those with two measurements, a two-way ANOVA was used. Both ANOVAs were run with Bonferroni *post hoc* test. In addition, a subgroup analysis was performed, according to the response in the antioxidant capacity and concentration of total phenols in serum: increase versus no increase. For this, the delta (agraz minus placebo) was calculated and transformed into a qualitative variable, where the positive values were considered increase and the negative or zero values not increase. Then ANOVAs were performed with analysis of multiple comparisons by Bonferroni test. All statistical analyses were performed in the Statistical Package for the Social Sciences software for Windows, version 24.0^®^ and graphics in GraphPad Prism for Windows, version 7^®^.

## Results and Discussion

Participants maintained their usual diet and physical activity, and their body weight was constant throughout the study. Adherence to the study was greater than 80% for men and 98% for women. Thus, the following results were not associated with weight loss, changes in the composition (quantity and quality) of the diet, or changes in physical activity.

In the population admitted to the study, 7.7% met five MS criteria, and 17.3% had four criteria. The most prevalent components of MS according to gender were abdominal obesity in women and elevated triglycerides in men, similar to the results reported by other authors.^43,44^ Mild adverse effects (i.e., heartburn) after the consumption of agraz were reported by two subjects, but without any serious consequence to continue in the study.

At the end, 15.4% of men and 26.9% of women finished the study without MS. Systolic blood pressure (SBP), triglycerides, and high-density lipoprotein cholesterol (HDL-c) were the most responsive components. We observed that women responded better and/or have a greater adherence. Other intervention studies have reported that women responded better to changes in diet than men.^45,46^

The components of the MS were analyzed over time and according to time × treatment and time × treatment × gender interactions. No differences were observed in physical activity and diet between placebo and agraz periods for each gender; however, differences between genders were observed (data no shown). Although no significant differences were found in the interactions, differences were observed over time in variables such as SBP, glucose, and HDL-c levels. For example, a reduction in BP was observed in the entire group (men + women; Table 1), which has been shown to be one of the most sensitive variables to change in intervention studies, compared to other components of MS.^47^ This has been attributed to the ability of polyphenols to increase the bioavailability of vasodilators such as nitric oxide.^12^ It is important to note that the values of diastolic BP were reduced in the period of agraz consumption, mainly in men by 2.7%; although this reduction was not statistically significant, it is clinically important given the risk factors of this population.^48^

**Table 1.** Effects of Agraz on Metabolic Syndrome Components According to Gender

Parameters		Men		Women		p^a^	p^b^	p^c^
	Treatment	Baseline	Final	Baseline	Final			
WC (cm)	Placebo	106 ± 1.6	106 ± 1.8	102 ± 1.8	102 ± 1.9	0.491	0.828	0.911
	Agraz	107 ± 1.7	106 ± 1.7	102 ± 1.8	102 ± 1.9			
SBP (mmHg)	Placebo	124 ± 2.1	123 ± 1.9	118 ± 2.6	115 ± 2.5	0.017^*^	0.982	0.629
	Agraz	124 ± 2	123 ± 1.8	118 ± 2.5	116 ± 2.3			
DBP (mmHg)	Placebo	76 ± 1.5	75 ± 1.3	76 ± 1.7	75 ± 1.6	0.001^*^	0.605	0.418
	Agraz	77 ± 1.7	75 ± 1.3	76 ± 1.7	75 ± 1.7			
Triglycerides (mg/dL)	Placebo	240 ± 16	235 ± 14	204 ± 15	189 ± 10	0.379	0.574	0.884
	Agraz	237 ± 15	231 ± 16	209 ± 19	203 ± 17			
Glucose (mg/dL)	Placebo	98 ± 1.5	97 ± 1.2	93 ± 1.2	96 ± 1.7	0.049^*^	0.994	0.058
	Agraz	96 ± 1.5	98 ± 1.6	96 ± 1.6	96 ± 1.7			
HDL-c (mg/dL)	Placebo	37 ± 1.2	39 ± 1.0	41 ± 1.1	41 ± 1.1	0.034^*^	0.927	0.613
	Agraz	37 ± 0.9	38 ± 1.2	41 ± 1.2	41 ± 1.3			

ANOVA of repeated measurements with multiple comparison Bonferroni test.

^*^ Significance <0.05.

^a^ Time.

^b^ Interaction time × treatment.

^c^ Interaction time × treatment × gender.

ANOVA, analysis of variance; DBP, diastolic blood pressure; HDL-c, high-density lipoprotein cholesterol; SBP, systolic blood pressure; WC, waist circumference.

The concentration of phenols had a tendency to increase after agraz consumption, both in men and women (Table 2). Studies with other *Vaccinium* species have shown increases in the content of phenols in people with MS.^12,49,50^ When analyzing by gender, women increased their antioxidant capacity, measured by ferric reducing antioxidant power, by 1.3%, while in men there was no increase. Although this difference could be considered minimal, other studies have reported differences between genders regarding antioxidant status,^33,51^ which might be attributed to the hormonal component.^52^

**Table 2.** Effects of Agraz on Antioxidant Capacity and Oxidative and Inflammatory Markers in Men and Women with Metabolic Syndrome

	Men		Women		p^a^	p^b^
	Placebo	Agraz	Placebo	Agraz		
Phenols (mg AG/L)	235 ± 7	239 ± 6	313 ± 13	328 ± 7	0.327	0.586
FRAP (μM Trolox/L)	696 ± 19	696 ± 21	632 ± 17	640 ± 26	0.955	0.943
NFκB (abs)	0.12 ± 0.0	0.12 ± 0.0	0.11 ± 0.0	0.11 ± 0.0	0.954	0.154
Insulin (mUI/L)	27 ± 2	25 ± 2.3	21 ± 1.9	19 ± 1.7	0.432	0.974
HOMA-IR	6.7 ± 2.9	6.6 ± 2.9	5 ± 2.4	4.6 ± 2	0.752	0.646
hs-CRP (mg/L)	3.7 ± 0.2	3.3 ± 0.2	4.5 ± 0.4	3.8 ± 0.3	0.108	0.505
TBARS (μM MDA)	1.2 ± 0.08	1.2 ± 0.07	0.8 ± 0.06	0.8 ± 0.07	0.408	0.207
oxLDL (ng/mL MDA)	341 ± 33	306 ± 32	331 ± 23	332 ± 23	0.652	0.376

Two-way ANOVA with multiple comparison for Bonferroni test.

^a^ Treatment.

^b^ Interaction treatment × gender.

FRAP, ferric reducing antioxidant power; HOMA-IR, homeostatic model assessment of insulin resistance; MDA, malondialdehyde; oxLDL, oxidized low-density lipoprotein; TBARS, thiobarbituric acid reactive substances.

Another interesting result was the decrease in the oxidative marker oxLDL by 11% in men, when they consumed agraz, compared to women (*p* = 0.376), in whom no considerable changes were observed (only 0.3% decrease, Table 2). Several studies have found beneficial effects of blueberries in oxidative markers,^22,53,54^ but the authors included only one gender or included both men and women but did not analyze effects separately, as in this study.

It is important to note that insulin levels and insulin resistance measured by HOMA-IR did not show significant (*p* = 0.974 and *p* = 0.646, respectively) differences between the genders after agraz consumption, which is in agreement with those reported by various studies.^29,55,56^ After agraz consumption, compared to placebo, there was a notable reduction in women compared to men both in insulin (11% vs. 8%, respectively) and in HOMA-IR (8.7% vs. 1.5%, respectively; Table 2).

Regarding the effects of agraz consumption on inflammation markers, hs-CRP levels decreased by 12.1% and 18.4%, in men and women, respectively (*p* = 0.505). This effect has been attributed to changes in endothelial function such as the increase in vasodilators, for example, nitric oxide, which has been associated with reductions in inflammatory markers.^57^ Although the differences presented by gender in inflammation were not significant, a greater reduction in women could be noted, mainly due to the narrow relationship between female hormones and the production of nitric oxide.^58,59^

**FIG. 1.**
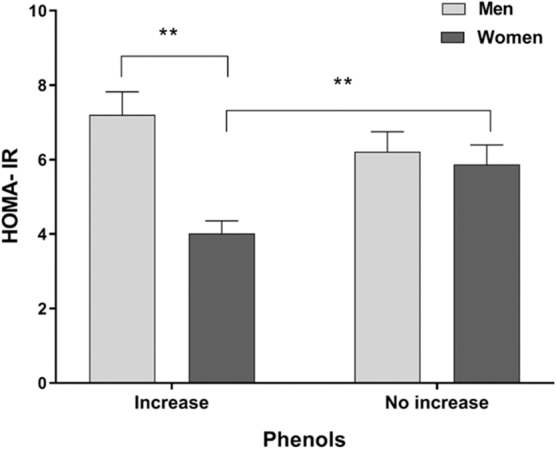
Comparative effects of agraz consumption on insulin resistance between participants who increased versus those with no increase in their phenol content and by gender in both conditions. Two-way ANOVA with diet consumption correction. *p < 0.05; **p < 0.01. ANOVA, analysis of variance; HOMA-IR, homeostatic model assessment of insulin resistance.

**FIG. 2.**
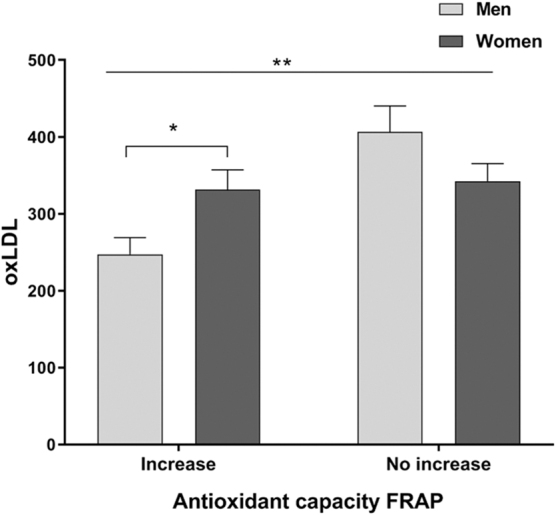
Comparative effects of agraz consumption on oxLDL concentrations between participants who increased versus not increased their antioxidant capacity (measured by FRAP) and by gender in both conditions. Two-way ANOVA with diet consumption correction. *p < 0.05; **p < 0.001. FRAP, ferric reducing antioxidant power; oxLDL, oxidized low-density lipoprotein.

Given the observed increase in the content of total phenols in both genders and the increase in antioxidant capacity in women after agraz consumption, a subgroup analysis was performed evaluating those who responded increasing their total phenol levels and their antioxidant capacity versus those who did not increase these variables after consuming the nectar of agraz. The results showed significant differences between genders for insulin resistance (Fig. 1) and the oxidative marker oxLDL (Fig. 2).

When analyzing the response on insulin resistance, differences were observed between men and women who increased their phenol content (*p* = 0.005, Fig. 1). The findings showed that the increase in phenols after consuming agraz in men did not impact statistically (*p* = 0.208) the changes in the HOMA-IR levels; while in women, there was a significant decrease in insulin resistance (*p* = 0.002) in those who increased their total phenol content, compared to those with no increase in this variable (Fig. 1). Studies with cranberries have shown increasing insulin sensitivity,^21,60^ but these studies do not analyze gender differences. Given that men have greater visceral adiposity than women, little protective effect of estrogen, reduction in insulin sensitivity, and low levels of adiponectin,^61,62^ this could explain to some extent the more promising results presented in women compared to men.

Subgroup analyses also showed that those who increased their antioxidant capacity after consuming agraz presented 29% significantly lower levels of oxLDL compared to those who did not increase their antioxidant capacity (291 ± 18 vs. 375 ± 21; *p* = 0.003). In addition, there was a statistically significant difference (*p* = 0.008) on this effect between genders, as shown in Figure 2. Studies with bilberries have shown beneficial effects on lipid peroxidation, mainly attributed to the increase in antioxidant capacity and its consequences on oxidative stress.^22,54^ This finding was also observed in the present study, for those who presented better antioxidant capacity (men) with a greater reduction in their levels of peroxidation measured by oxLDL. Although women presented higher values of phenols compared to men, this was not reflected in their levels of antioxidant capacity nor in reductions on oxidative marker levels.

## Conclusions

The dose of agraz provided in this study increased total phenols and antioxidant capacity in the serum of participants with MS. This increase was greater in women than in men. Although there were no statistical differences in the primary variables according to the initial design, as evidenced in Tables 1 and 2, the subgroup analysis comparing those who increased versus those who did not increase their phenols and antioxidant capacity after agraz consumption showed even more differential responses between genders, with a significant reduction in insulin resistance in those women who increased their phenols and a significant reduction in oxLDL in those men who increased their antioxidant capacity. These results confirm not only the effect of agraz on insulin resistance and lipid peroxidation associated to the improvement in antioxidant status but also the marked gender differences on these effects in adults with MS. Therefore, gender should be considered both to evaluate the risk and to determine the more appropriate type of therapy or intervention.

## Abbreviations Used

ANOVA: analysis of variance
BP: blood pressure
DBP: diastolic blood pressure
ELISA: enzyme-linked immunosorbent assay
FRAP: ferric reducing antioxidant power
HDL-c: high-density lipoprotein cholesterol
HOMA-IR: homeostatic model assessment of insulin resistance
hs-CRP: high-sensitive C-reactive protein
MDA: malondialdehyde
MS: metabolic syndrome
NFκB: nuclear factor enhancer of the kappa light chains of activated B cells
oxLDL: oxidized low-density lipoprotein
ROS: reactive oxygen species
SBP: systolic blood pressure
TBARS: thiobarbituric acid reactive substances
WC: waist circumference
